# Comparative Metabolite Profiling and Antiproliferative Characterization of Lab-Acclimatized and Wild Green Seaweed *Acrosiphonia orientalis* to Reveal Its Nutraceutical Potential

**DOI:** 10.3390/foods15071252

**Published:** 2026-04-06

**Authors:** Deepesh Khandwal, Jalak N. Maniar, Shruti Kumari, Pratishtha Menaria, Avinash Mishra

**Affiliations:** 1 Division of Applied Phycology and Biotechnology, CSIR-Central Salt and Marine Chemicals Research Institute, Bhavnagar 364002, India; 2 Academy of Scientific and Innovative Research (AcSIR), Ghaziabad 201002, India

**Keywords:** *Acrosiphonia*, functional food, cultivation, metabolomics, nutraceutical, antiproliferative activity, gene expression analysis

## Abstract

The increasing demand for different value-added products from natural seaweeds requires a sustainable cultivation method for the regular supply of biomass and to safeguard the natural ecosystem from overexploitation. This study evaluated laboratory acclimatization of the green seaweed *Acrosiphonia orientalis* (DGR: 2.71 ± 0.21%; GPP: 12.55 ± 0.1 mg O_2_ L^−1^ day^−1^), followed by a comparative evaluation of its physicochemical and biochemical characteristics, metabolite profile, and antiproliferative activity compared with naturally harvested seaweed. Metabolite profiling identified 47 compounds exhibiting differential accumulation patterns, with the natural specimens enriched in omega-3 polyunsaturated fatty acids, including docosahexaenoic acid, and the laboratory-acclimatized specimens exhibited elevated arachidonic acid levels. Amino acid profiling revealed higher concentrations of essential and non-essential amino acids in the natural specimens, with prominent levels of phenylalanine and aspartic acid, while the lab-acclimatized specimens were enriched in isoleucine, methionine, proline, and cysteine. The lab-acclimatized specimens exhibited significantly enhanced water absorption (WSC: 6 ± 0.25 mL/g DW; WHC: 2.68 ± 0.11 g/g DW) and higher total sugar (47.11 ± 0.52% Glc eq. DW) and phenolic contents (51.28 ± 0.54 mg GAE g^−1^ extract), while the natural specimens had a superior oil-holding capacity (OHC: 1.8 ± 0.12 g/g DW); higher total flavonoid (123.62 ± 2.97 mg Q g^−1^ extract), protein (5.11 ± 0.36 µg BSA eq/mg DW), and chlorophyll contents (8.82 ± 0.58 mg/L); and higher antioxidant activities (ABTS-EC_50_: 67.33 ± 0.97 μg/mL extract). The mineral analysis revealed distinct elemental profiles, with enrichment of sodium, magnesium, and calcium in the lab-acclimatized specimens and a more favorable Na/K ratio (0.14 vs. 0.78) in the natural specimens. Of note, extracts from both seaweeds exhibited significant dose-dependent antiproliferative activity against HeLa cervical cancer cells (Wild EC_50_: 118.63 ± 14.14 µg/mL extract; lab EC_50_: 153.35 ± 10.18 µg/mL extract), suppressed colony formation in soft agar assays, induced nuclear condensation (based on Hoechst staining), and modulated the expression of key oncogenes (upregulating *NDRG1*, *TP53*, and *CASP3* and downregulating *BCL2*, *MYC*, and *CCND1*). Collectively, this study provides an approach to acclimatize *A. orientalis* that may be utilized for developing a cultivation method. Moreover, this green seaweed has a great potential to be used for nutraceutical and functional food applications.

## 1. Introduction

Marine macroalgae represent significant sources of structurally diverse bioactive metabolites with demonstrated pharmacological properties. *Acrosiphonia orientalis*, a tropical filamentous chlorophyte, has attracted scientific interest due to its distinctive secondary metabolite profile and its documented biological activities. However, systematic cultivation of *A. orientalis* under artificial conditions remains unexplored [1,2,3]. This green seaweed also possesses significant potential as a marine bioresource owing to its abundant antioxidants, phenolic compounds, flavonoids, sulfated polysaccharides, essential amino acids, and fatty acids. These diverse bioactive constituents make it particularly suitable for nutraceutical and functional food development [4,5]. In addition, sulfated polysaccharides derived from green seaweed have demonstrated a wide range of biological benefits, including antioxidant, anti-inflammatory, and anticancer activities [5,6]. Building on these properties, recent research on *A. orientalis* has utilized deep eutectic solvent extraction and nano-emulsion techniques to produce extracts with increased effectiveness against various human cancer cell lines [7]. *A. orientalis*, along with other marine macroalgae, synthesizes numerous bioactive substances that have garnered considerable attention for their pharmaceutical applications. The secondary metabolic pathways in these organisms yield compounds with proven efficacy against various pathological conditions, including malignant cell proliferation, bacterial infections, fungal diseases, viral pathogens, inflammatory disorders, and oxidative-stress-related ailments [3,8,9]. 

There are numerous practical obstacles to the traditional dependence on naturally harvested algal materials, including variations in chemical composition due to environmental factors, restricted seasonal availability, concerns regarding ecosystem conservation, and challenges in maintaining uniform quality standards [10,11]. Managing cultivation approaches offers promising solutions by providing controlled growth environments that enable predictable biomass production, stable compound concentrations, reduced environmental impact, and year-round harvesting capabilities [12]. Nevertheless, changes in environmental conditions often alter the biological functions and biochemical production in marine species, requiring comprehensive comparative studies to understand these effects [13].

Once harvested from its natural habitat, characterization of *A. orientalis* has highlighted its unique metabolite profile and its associated biological activities, particularly its antioxidant properties [3]. However, the metabolic and bioactive response of *A. orientalis* when grown under controlled conditions are unknown. It remains unclear whether a controlled culture environment may trigger shifts or alter the biosynthesis of bioactive metabolites. The absence of standardized growth methods represents a significant barrier to scaling up the production of this seaweed. To successfully cultivate algae on a commercial scale, it is necessary to fine-tune numerous environmental factors that affect how the organisms grow and produce beneficial compounds [14,15]. These variables include light intensity and duration, nutrient composition, temperature control, salt concentration, and pH, all of which play essential roles in determining the growth rates, metabolic processes, and formation of valuable secondary compounds [11,16].

The present study aimed to address this gap by comparing naturally harvested *A. orientalis* (WildAO) with lab-acclimatized *A. orientalis* (LabAO) across various parameters, including the daily growth rate (DGR), photosynthesis (gross primary productivity (GPP) and the respiration rate), and the chlorophyll content. This study tested the hypothesis that laboratory acclimatization induces specific changes in the lipid and phenolic compositions, which modulates the antiproliferative potency of *A. orientalis*. To test this hypothesis, the nutritional profile of WildAO and LabAO was compared, including an analysis of the physicochemical and biochemical characteristics; the metabolite, fatty acid, amino acid, and mineral contents; and antioxidant activities. Additionally, antiproliferative and soft gel agar assays as well as gene expression analyses were performed to compare the potential antioxidant activity of the WildAO and LabAO extracts.

## 2. Materials and Methods

### 2.1. Sample Collection and Lab-Acclimatization

*A. orientalis* specimens were collected in March 2024 from the coastal region of Diu, Gujarat, India (20°42′09.5″ N, 70°55′02.6″ E). At the time of the sampling, the water temperature and salinity were 26–27 °C and 30–32 ppt, respectively, with 11–12 h of daylight [17]. After harvesting, the samples were rinsed thoroughly with filtered seawater (salinity = 30 ppt) to eliminate the surface-associated epiphytes and debris. A portion of the collected biomass was air-dried under shade at ambient temperature for 20 days and subsequently stored in airtight containers. The remaining cleaned biomass was kept in a glass tank (16 L) with a biomass density of 10 g/L (fresh weight) in seawater (salinity = 30–32 ppt, pH = 7.8), which was enriched with 0.01% (*w*/*v*) potassium phosphate (NaH_2_PO_4_) and sodium nitrate (NaNO_3_). The culture tank was maintained at 25–26 °C under a 12 h photoperiod with white light illumination at an intensity of 25 μmol photons m^−2^ s^−1^ [17]. The seawater was refreshed weekly, and specimens were gently cleaned to prevent biofouling. For the first 4 months, the health and growth of the biomass were monitored periodically. At the end of the fifth month of acclimatization, seaweed was harvested and subjected to shade-drying at ambient temperature for 20 days. The dried WildAO and LabAO samples were finely powdered using a ball mill and stored in −80 °C for further use.

### 2.2. Daily Growth Rate Estimation

Apical segments exhibiting healthy frond were selected for further acclimatization. From these, fragments (approximately 100 mg) were prepared in triplicate and transferred into 100 mL glass beakers containing 0.01% (*w*/*v*) NaH_2_PO_4_ and NaNO_3_ with salinity of 30 ppt in static conditions. The experimental units were maintained under controlled conditions at 25 °C with a 12 h photoperiod (25 μmol photons m^−2^ s^−1^). The culture medium was replenished weekly, and the thalli were gently cleaned using a soft brush to minimize microbial contamination and surface fouling. Following a 15-day incubation period, the daily growth rate (DGR) of the fragments was calculated based on changes in biomass using the formula DGR (% per day) = (*t*ln(*W_t_*) − ln(*W*_0_)) × 100, where *W*_0_ is the initial fresh weight, *W_t_* is the final fresh weight, and *t* is the cultivation time in days [18].

### 2.3. Calculation of Primary Productivity

A total of 100 mg of fresh *A. orientalis* fronds was added to 250 mL glass bottles containing autoclaved seawater. Two experimental conditions were established: one set of bottles (in triplicate) was incubated in complete darkness, while the other set was maintained under illuminated conditions (25 μmol photons m^−2^ s^−1^, 12 h light cycle) at 25 °C for 3 days. In the dark-incubated bottles, only respiratory activity occurred, whereas in the light-exposed bottles, both photosynthetic and respiratory processes were active. The rate of respiration (R) was determined by quantifying the decrease in dissolved oxygen (DO) concentration in the dark-incubated bottles. Net primary productivity (NPP) was assessed by measuring the increase in DO concentration by probe sensor in the light-exposed samples. Gross primary productivity (GPP) was then calculated as the sum of NPP and R (GPP = NPP + R), representing the total photosynthetic output, expressed as milligrams of oxygen per liter per day (mg O_2_ L^−1^ day^−1^) [19].

### 2.4. Physicochemical Analysis

#### 2.4.1. Assessment of Water Swelling and Water Retention Properties

To assess the water swelling capacity (WSC; mL g^−1^ DW), 100 mg of crude seaweed powder (W) was suspended in 10 mL of distilled water [20]. The initial volume of the suspension (V_i_) was recorded, and the mixture was incubated at 25 °C for 24 h at ambient conditions. After incubation, the final volume (V_o_) was measured. The water-holding capacity (WHC; g g^−1^ DW) was evaluated by dispersing 100 mg of dry crude seaweed (W_D_) in 10 mL of distilled water in preweighed centrifuge tubes [21]. The mixtures were incubated at 37 °C for 24 h with shaking. After incubation, the samples were centrifuged at 3000 *g* for 25 min at ambient temperature. The supernatant was discarded, and the hydrated residue was weighed (W_W_) [5]. The WSC and WHC were calculated using the following equations:

$$WSC\;\left(mL\;g^{-1}\;DW\right)=\frac{V_{o}\;-\;V_{i}}{W}\;WHC\;\left(g\;g^{-1}\;DW\right)=\;\frac{W_{W}\;-\;W_{D}}{W_{D}}$$

#### 2.4.2. Determination of Oil-Holding Capacity

To measure the oil-holding capacity (OHC), 1 g of crude seaweed powder was thoroughly mixed with 10 mL of canola oil (density = 0.91 g mL^−1^) in a preweighed centrifuge tube [22]. The mixture was vortexed briefly and incubated at ambient temperature for 30 min with mild shaking, followed by centrifugation at 2500 *g* for 30 min. After carefully decanting the supernatant, the weight of the retained oil-bound residue was recorded [23]. The OHC was calculated using the following equation and is expressed as grams of oil bound per gram of dry weight (g g^−1^ DW):

$$OHC\;\left(g\;g^{-1}\;DW\right)=\frac{Wet\;weight\;residue-Sample\;dry\;weight}{Sample\;dry\;weight}$$

### 2.5. Biochemical Composition Analysis

#### 2.5.1. Moisture Content, Dry Weight, and Mineral Composition

Fresh WildAO and LabAO samples were oven-dried at 60 °C for 1 week. The moisture content was determined based on the difference between the initial fresh mass and the final dry mass. To determine the ash content, 100 mg of powdered dry material was incinerated in a muffle furnace at 550 °C for 24 h to achieve complete mineralization. The ash residue was subsequently subjected to acid digestion using a mixture of three acids (HNO_3_:HCl:H_2_O_2_, 1:2:3, *v*/*v*) at 80 °C, continued until brown fumes ceased to be released, indicating complete digestion. The digestate was diluted in 10 mL of deionized water, centrifuged at 14,000 rpm, and the supernatant was used for mineral and elemental profiling through inductively coupled plasma mass spectrometry (ICP-MS, PerkinElmer, Shelton, CT, USA) following standardized protocols [24,25].

#### 2.5.2. Total Protein and Carbohydrate Quantification

Fifty milligrams of seaweed samples that had been stored at −80 °C were subjected to extraction with a 10 mL solvent (methanol: isopropanol: water, 5:3:2, *v*/*v*/*v*) by sonication for 20 min, followed by incubation for 1 h at room temperature (26–28 °C) with a shaking of 120 rpm. The supernatant was collected by centrifugation at 10,000 rpm for 15 min at 4 °C. The collected supernatant was stored at −20 °C and utilized for biochemical—the total protein, total carbohydrate content, total phenolic content (TPC) and total flavonoid content (TFC)—and antioxidant activity assays.

The total protein content was evaluated using the Bradford method [26], wherein 1 mg/mL aliquots were reacted with an equal volume of Bradford reagent and incubated at room temperature for 10 min. The protein concentration was determined spectrophotometrically at 595 nm using a bovine serum albumin (BSA) standard curve and expressed as micrograms of BSA equivalents per milligram dry weight (µg BSA eq./mg DW) [26]. The total carbohydrate content was quantified using the phenol–sulfuric acid method. Samples (1 mg/mL) were treated with 50 µL of 80% phenol and 2 mL of concentrated sulfuric acid, followed by incubation for 20 min. Absorbance was measured at 490 nm and sugar content was calculated against a glucose (Glc) standard curve. The results are expressed as percent Glc equivalent per dry weight (% Glc eq./DW) [27,28].

#### 2.5.3. Total Lipid Content

Gravimetric analysis of total lipids was conducted using the method of Bligh and Dyer [29]. Approximately 500 mg of powdered sample was subjected to extraction with 5 mL of chloroform:methanol (2:1, *v*/*v*) and incubated in the dark for 24 h. The extract was filtered and washed with 1 mL of 0.9% (*w*/*v*) sodium chloride for phase separation. The organic (chloroform) phase was collected, dried using a hot plate, and the remaining lipid content was quantified gravimetrically [25,29].

#### 2.5.4. Total Phenolic and Flavonoid Content

The TPC was determined with the Folin–Ciocalteu method [30]. Extracts (250 µL, 1 mg/mL) were reacted with 500 µL of 2 M Folin–Ciocalteu reagent and 2 mL of 20% Na_2_CO_3_, vortexed, and incubated at 40 °C for 30 min in the dark. After centrifugation at 9000 *g* for 2 min, the absorbance was measured at 760 nm. Quantification was done using a gallic acid standard curve, and results were presented as milligrams of gallic acid equivalents per gram dry extract (mg GAE/g extract). The TFC was determined via a colorimetric method using the aluminum chloride (AlCl_3_) method [31]. Extracts were sequentially treated with 5% NaNO_2_ (300 µL), 10% AlCl_3_ (300 µL), and 1 M NaOH (2 mL), with absorbance measured at 510 nm. The TFC was determined using a quercetin standard and is expressed as milligrams of quercetin equivalents per gram dry extract (mg Q/g extract) [28].

#### 2.5.5. Chlorophyll and Carotenoid Estimation

Pigments were analyzed after subjecting samples to extraction using chilled acetone [32]. Approximately 20 mg of dry sample was added to 1 mL of chilled acetone and incubated at 4 °C for 30 min in the dark. After centrifugation at 9000 *g* for 15 min at 4 °C, the absorbance of the supernatant was measured at 665, 664, 647, and 461 nm. Total chlorophyll and carotenoid contents were calculated using the following equations:

$$Total\;chlorophyll\;content\left(mg/L\right)=\left(17.90\;A_{647}\right)+\left(8.08\;A_{665}\right)$$

$$Total\;carotenoid\;content\left(mg/L\right)=\left[A_{461}-\left(0.046\;A_{664}\right)\right]\times 4$$

### 2.6. Total Antioxidant Capacity and Radical Scavenging Activity

Antioxidant activity was assessed based on the 2,2′-azino-bis(3-ethylbenzothiazoline-6-sulfonic acid) (ABTS) and 2,2-diphenyl-1-picrylhydrazyl (DPPH) radical scavenging assays. ABTS radicals were generated by mixing 7 mM ABTS solution with 2.45 mM potassium persulfate and incubating in the dark for 12–16 h. The solution was diluted to an absorbance of 0.70 ± 0.02 at 734 nm. The WildAO and LabAO extracts (25–100 µg/mL) were incubated with 1 mL of ABTS solution at room temperature for 90 min. Absorbance was measured at 734 nm and compared to an ascorbic acid standard [33]. A DPPH solution in methanol was adjusted to an initial absorbance at 517 nm of 0.98 ± 0.02. The WildAO and LabAO extracts were incubated with 1 mL of DPPH solution at room temperature in the dark for 1 h, and absorbance was recorded at 517 nm. The radical scavenging percentage was calculated relative to a reference standard [4,34]. In parallel, blanks were runs with seaweed extract and reagents but without radicals. The absorbance of the blanks was subtracted from the sample wells to account for background absorbance.

### 2.7. Primary Metabolites Profiling

For primary metabolite profiling, 100 mg of powdered sample was subjected to extraction with ice-cold methanol with ribitol (0.1 mg/mL) as the internal standard [35,36]. The mixture was incubated at 70 °C for 30 min, and then centrifuged at 11,000 *g*. The supernatant was mixed with chloroform and water, vortexed, centrifuged again, and the upper aqueous phase was collected and dried. Derivatization was performed by incubating the samples with methoxyamine hydrochloride (80 µL) at 65 °C for 90 min, followed by the addition of 120 µL of MSTFA + 1% TMCS and a second incubation at 65 °C for 2 h. The derivatized sample was analyzed via GC–MS (QP-2010, Shimadzu, Kyoto, Japan) using an autosampler and RTX 5MS capillary column (60 m long, 0.25 mm in diameter, and 0.50 µm thick). The sample was analyzed with helium gas as carrier (with a flow rate of 2 mL min^−1^). Ionization was performed by electron impact (70 eV), and the ion source and quadrupole were kept at 200 °C [37]. The initial column temperature was 80 °C for 2 min, and then increased to 315 °C at 10 °C min^−1^. The injection volume was 1 μL, the run time was 40 min, and the temperature was 250 °C [33]. The metabolites were identified by comparison with NIST database (80% threshold score) and quantified based on normalization to ribitol [25,38].

### 2.8. Fatty Acid Methyl Ester Analysis

Lipid profiling was performed via fatty acid methyl ester (FAME) analysis [29]. Lipid extracts (10 mg) were trans-esterified with 1 mL of 1% NaOH in methanol at 55 °C for 15 min, followed by 2 mL of 5% HCl in methanol for an additional 15 min. FAMEs were extracted with hexane, dried under nitrogen, and reconstituted in 150 µL hexane. Analysis was performed by GC–MS, and fatty acids were identified using a commercial C4–C24 FAME standard mixture (Sigma-Aldrich, St. Louis, MO, USA). Quantification was based on peak area normalization [28].

### 2.9. Amino Acid Profiling

The amino acid composition was analyzed by hydrolyzing 100 mg of freeze-dried sample in 200 μL of 6N HCl supplemented with 1% phenol. The hydrolysis was carried out at 110 °C for 24 h, followed by drying at 100 °C for an additional 24 h within sealed glass vials. The resulting hydrolysate and amino acid standard solution (10 μL, AAS18, Sigma-Aldrich) was purged with nitrogen gas and then sealed to minimize oxidation. A prederivatization step involved dissolving the dried content in 500 μL of an ethanol: water: triethylamine (TEA) mixture (2:2:1, *v*/*v*/*v*), from which 20 μL was removed, vortexed, and vacuum-dried. Derivatization was performed by adding 500 μL of ethanol: water: TEA: PITC (7:1:1:1, *v*/*v*/*v*/*v*), and the mixture was incubated for 20 min at room temperature. After evaporation, the residue was reconstituted in 400 μL of 5 mM sodium phosphate buffer (pH 7.4) containing 5% acetonitrile. The solution was centrifuged, and 120 μL of supernatant was filtered through a 0.45 μm membrane prior to high-performance liquid chromatography (HPLC, Waters Alliance, Milford, MA, USA). Chromatographic separation was performed using a C18 reversed-phase column (Luna, 4.6 × 150 mm, 5 μm, Phenomenex, Torrance, CA, USA) with a 40 μL injection volume and a 1 mL/min flow rate. The eluents used were buffers A (150 mM sodium acetate, 6% acetonitrile, 0.05% TEA, pH 6.4) and B (acetonitrile: water, 6:4, *v*/*v*). Detection was carried out at 254 nm, and amino acids were identified by comparing retention times with those of standards (A6407, Sigma-Aldrich). Quantification was based on peak area normalization [28,33].

### 2.10. Cell Culture, Antiproliferative Activity and Nuclear Fragmentation Assay

HeLa cells, a human cervical carcinoma-derived cell line, were obtained from the National Centre for Cell Science (NCCS, Pune, India) and confirmed to be *Mycoplasma*-free prior to culture. Cells were cultured in MEM medium (Minimum Essential Medium Eagle w/Earle’s salts, 2 mM L-Glutamine, 1 mM Sodium pyruvate, NEAA and 1.5 g per liter Sodium bicarbonate; AL047S, Hi-Media, Maharashtra, India) supplemented with 10% fetal bovine serum (FBS) and a 1% antimycotic mixture, maintained at 37 °C in a humidified incubator with 5% CO_2_. WildAO and LabAO samples were subjected to extraction overnight with 60% (*v*/*v*) aqueous methanol for 12 h at 26 °C. Then the samples were lyophilized to evaporate solvent and dissolved in phosphate-buffered saline (PBS). To test the antiproliferative activity of the extracts, HeLa cells were seeded into 96-well plates at a density of 1 × 10^5^ cells/mL and allowed to adhere for 24 h. Thereafter, cells were treated with 25–100 μg/mL of the WildAO or LabAO extracts; PBS was used as the vehicle control. After treatment for 24 h, cell viability was evaluated using an MTT-based in vitro toxicology assay kit (TOX1-1KT, Sigma-Aldrich), with absorbance recorded at 570 nm. The antiproliferative effect was calculated using standard equations [5,33].

$$\mathrm{Antiproliferative}\;\mathrm{activity}\;(\%)=100\times \left[1-\frac{\left({\mathrm{abs}}_{570(\mathrm{sample})}\right)-\left({\mathrm{abs}}_{690(\mathrm{blank})}\right)}{\left({\mathrm{abs}}_{570(\mathrm{control})}\right)-\left({\mathrm{abs}}_{690(\mathrm{blank})}\right)}\right]$$

To assess nuclear morphology, HeLa cells were seeded at 1 × 10^5^ cells/mL in 96-well plates and treated with the respective half-maximal effective concentration (EC_50_) of the WildAO or LabAO extract; PBS served as the negative control. After incubation for 24 h at 37 °C, Hoechst 33342 dye (10 μg/mL) was added to each well. Following incubation for 10 min incubation, nuclear condensation and fragmentation were visualized under a fluorescence microscope [33,39].

### 2.11. Soft Agar Colony Formation Assay

Anchorage-independent growth of HeLa cells was evaluated in 6-well plates. The bottom layer comprised 2.0 mL of 0.6% agar mixed with 2× complete medium, which was allowed to solidify. The top layer (4.0 mL) was prepared by suspending trypsinized HeLa cells (1 × 10^3^ cells/mL) in melted 0.3% agar mixed at ~42 °C with an equal volume of medium, then poured over the base layer. Cultures were incubated at 37 °C for 14 days with 100 μL of complete medium added twice a week. On day 14, the wells were treated with the EC_50_ of the WildAO or LabAO extract, and the cells were observed for another 14 days. The colony size was observed on days 21 and 28 under 5× magnification and analyzed using the ImageJ software ver. 1.x (NIH, Bethesda, MD, USA). An image of a hemocytometer (taken at 5× magnification) served as a scale to compare the treated and control groups [40].

### 2.12. Gene Expression Analysis

HeLa cells were seeded in 6-well plates at 1 × 10^5^ cells/mL and incubated for 24 h before treatment with the EC_50_ of the WildAO or LabAO extract. Total RNA was isolated using a kit (MO-BIO Laboratories, Carlsbad, CA, USA), followed by synthesis of cDNA using a commercial kit (Takara, Kyoto, Japan). The cDNA was used for real-time polymerase chain reaction (RT-PCR) to examine the expression of genes related to oxidative stress (*GPX1*), the cell cycle (*CCND1* and *MYC*), tumor suppression (*TP53* and *NDRG1*), apoptosis (*CASP3*), and cell survival (*BCL2*) [3,6]. *GAPDH* was used as a housekeeping (reference) gene (Supplementary Table S1). Each 25 μL reaction contained 12.5 μL of ready-to-use TB Green Premix Ex Taq II (RR820A, Takara), 1.0 μL of cDNA (100 ng), 1 μL each of forward and reverse primers (10 μM), and 9.5 μL milliQ water. The reactions were run on a CFX96 system (Bio-Rad, Hercules, CA, USA) with the following thermal cycling program: initial denaturation at 95 °C for 30 s; 40 cycles at 95 °C for 5 s and 60 °C for 30 s [3]. The relative fold gene expression (2^−ΔΔCt^) was calculated using the Livak method [41] to compare expression between the treated and non-treated (control) cells as follows:

$$Relative\;fold\;expression=2^{-\Delta \Delta Ct}$$

$$\Delta \Delta C_{t}=\Delta C_{t}\left(treated\right)-\Delta C_{t}\left(untreated/control\right)$$

$$\Delta C_{t}\left(treated\right)=C_{t}\left(target\;gene\right)-C_{t}\left(refernce\;gene\;GAPDH\right)$$

$$\Delta C_{t}(untreated/control)=C_{t}\left(target\;gene\right)-C_{t}\left(refernce\;gene\;GAPDH\right)$$

### 2.13. Statistical Analysis

All experiments were performed in triplicates, and the results are expressed as the mean ± the standard error of the mean (SEM; *n* = 3). All experiments were performed in technical replicates, but biological triplicates were used to estimate the DGR and GPP. Statistical comparisons were conducted using one-way ANOVA followed by the *t*-test, with *p* < 0.05 considered to indicate a statistically difference. Different letters in the graphs and tables indicate statistically significant differences. Metabolomic data visualization and multivariate analyses, including heatmap generation, PCA, PLS-DA, and VIP score plots, were performed using MetaboAnalyst 6.0. Before statistical analysis, the metabolomic data were normalized by median of each sample, log10-transformed, and auto-scaled. The robustness and predictive power of the PLS-DA model were validated with indicators of statistical significance and a lack of overfitting considering Q^2^ ≥ 0.9 and a minimal R^2^–Q^2^ difference (≤0.1).

## 3. Results

### 3.1. Growth Parameters Estimation

**Figure 1:**
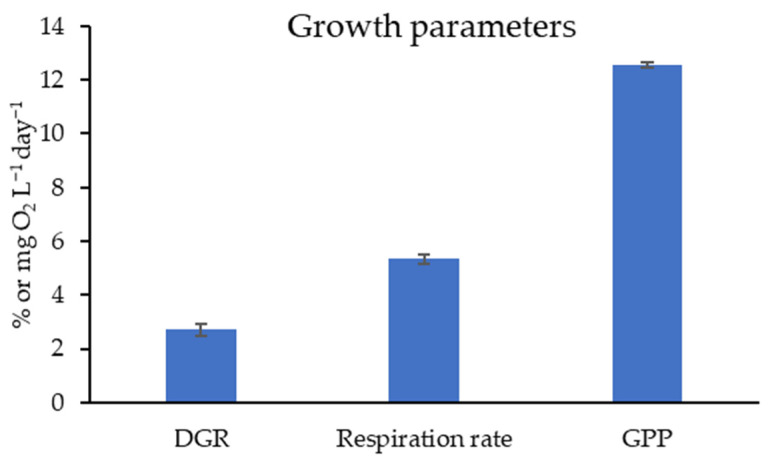
Growth parameter study of the lab-acclimatized *A. orientalis* (LabAO). DGR: daily growth rate (%); respiration rate (mg O_2_ L^−1^ day^−1^) and GPP: gross primary productivity (mg O_2_ L^−1^ day^−1^). Results are presented as the mean ± SEM (*n* = 3).

LabAO exhibited a DGR of 2.71 ± 0.21%, indicating steady biomass accumulation under controlled conditions (Figure 1). The respiration rate was 5.34 ± 0.17 mg O_2_ L^−1^ day^−1^, reflecting moderate metabolic activity. GPP was 12.55 ± 0.1 mg O_2_ L^−1^ day^−1^, demonstrating efficient photosynthetic performance.

### 3.2. Physicochemical Composition

**Figure 2:**
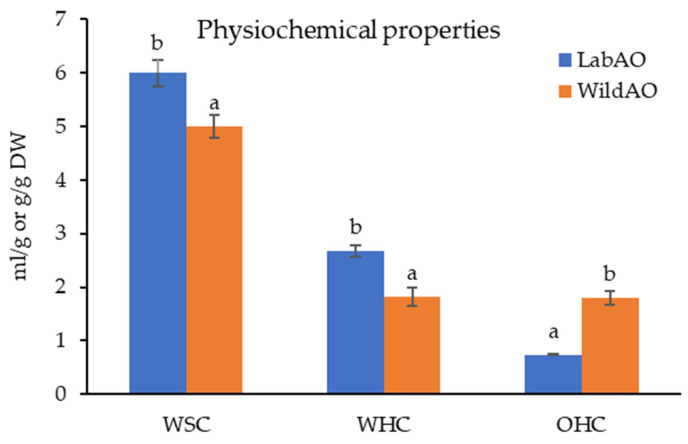
Physiochemical properties of the LabAO and WildAO biomass of *A. orientalis.* WSC: water swelling capacity (mL/g DW); WHC: water-holding capacity (g/g DW); and OHC: oil-holding capacity (g/g DW). Results are presented as the mean ± SEM (*n* = 3), with distinct letters denoting significant differences (*p* < 0.05), as determined by *t*-test.

The WSC was significantly higher for LabAO (6 ± 0.25 mL/g DW) compared to WildAO (5 ± 0.21 mL/g DW), indicating greater water absorption potential in LabAO (Figure 2). Similarly, WHC was higher for LabAO (2.68 ± 0.11 g/g DW) compared with WildAO (1.82 ± 0.18 g/g DW), suggesting enhanced water retention properties in the controlled environment-grown biomass. In contrast, OHC was higher for WildAO (1.8 ± 0.12 g/g DW) compared with LabAO (0.74 ± 0.02 g/g DW), indicating greater oil absorption in WildAO.

### 3.3. Comparative Biochemical Composition

**Figure 3:**
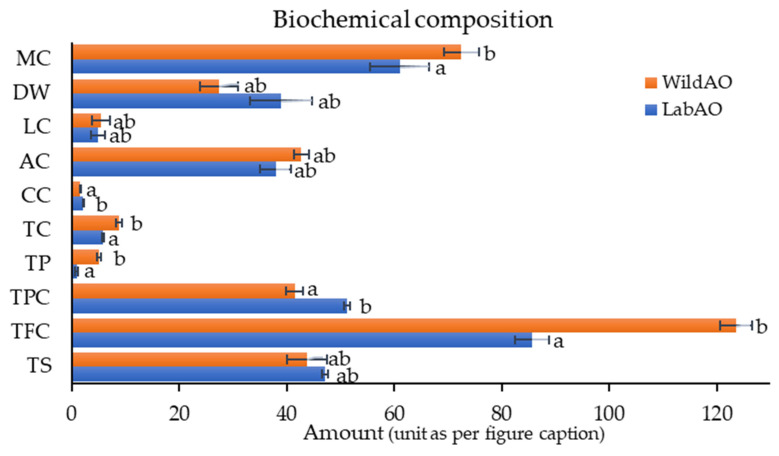
Biochemical compositions of the LabAO and WildAO specimens of *A. orientalis*. MC: moisture content (%); DW: dry weight (%); LC: lipid content (%); AC: ash content (%); CC: carotenoid content (mg/L); TC: total chlorophyll (mg/L); TP: total protein (µg BSA eq/mg DW); TPC: total phenolic content (mg GAE g^−1^ extract); TFC: total flavonoid content (mg Q g^−1^ extract); and TS: total sugar (% Glc eq. DW). Results are presented as the mean ± SEM (*n* = 3), with distinct letters denoting significant differences (*p* < 0.05), as determined by *t*-test.

The lab-acclimatized *A. orientalis* (LabAO) showed higher total sugar content (47.11 ± 0.52% Glc eq. DW) compared to WildAO (43.8 ± 3.76% Glc eq. DW) (Figure 3). In contrast, the TFC was higher for WildAO (123.62 ± 2.97 mg Q g^−1^ extract) compared with LabAO (85.62 ± 3.18 mg Q g^−1^ extract). The TPC was higher for LabAO (51.28 ± 0.54 mg GAE g^−1^ extract) relative to WildAO (41.53 ± 1.6 mg GAE g^−1^ extract). The protein content was substantially lower for LabAO (0.96 ± 0.27 µg BSA eq/mg DW) than for WildAO (5.11 ± 0.36 µg BSA eq/mg DW). The chlorophyll content followed a similar trend, with a higher concentration for WildAO (8.82 ± 0.58 mg/L) than LabAO (5.83 ± 0.18 mg/L). However, LabAO contained more carotenoids (2.19 ± 0.06 mg/L) than WildAO (1.63 ± 0.08 mg/L). The ash content was slightly lower for LabAO (37.95 ± 2.85%) compared with WildAO (42.7 ± 1.4%). The moisture content was notably higher for WildAO (72.51 ± 3.57%) compared with LabAO (61.04 ± 5.69%), reflecting fundamental differences in the water content between the cultivation methods. These differences highlighted how controlled conditions profoundly impacted on the biochemical composition, leading to a distinct profile for LabAO versus WildAO. Overall, the variation in the biochemical parameters underscores the metabolic adaptability of *A. orientalis* to its specific growth environment.

### 3.4. Antioxidant Activity

**Figure 4:**
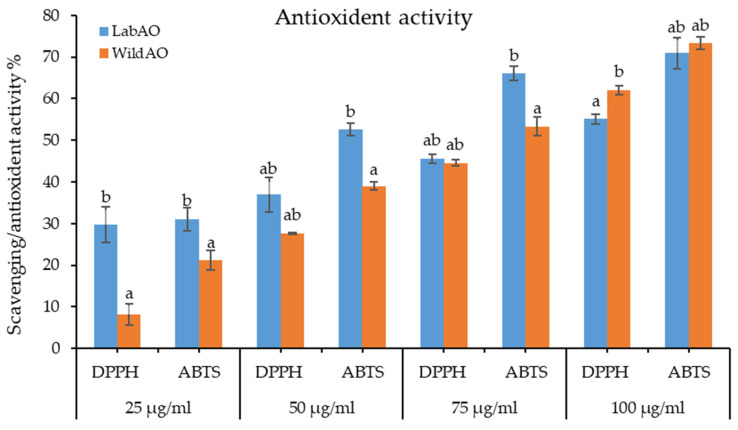
DPPH and ABTS radical scavenging-based antioxidant activity of the LabAO and WildAO extracts of *A. orientalis*, expressed in concentration gradient (25, 50, 75 and 100 μg/mL). DPPH: 2,2-diphenyl-1-picrylhydrazyl; ABTS: 2,2′-azino-bis (3-ethylbenzothiazoline-6-sulfonic acid. Results are presented as the mean ± SEM (*n* = 3), and different letters indicate a statistically significant difference (*t*-test *p* < 0.05).

The ABTS and DPPH radical scavenging assays were used to measure antioxidant activity (Figure 4). At the lowest tested concentration (25 μg/mL), WildAO extract exhibited significantly lower DPPH radical scavenging activity (8.12 ± 2.54%) compared to LabAO extract (29.7 ± 4.32%). However, at higher concentration (100 μg/mL), WildAO extract showed higher scavenging (62.1 ± 1.05%) compared to LabAO (55.12 ± 1.15%). The ABTS radical scavenging activity showed similar results; at 25 μg/mL, it was higher for the LabAO extract (31.01 ± 2.74%) compared with the WildAO extract (21.13 ± 2.33%), but this reversed at 100 μg/mL (WildAO = 73.29 ± 1.44%; LabAO = 70.95 ± 3.71%). The EC_50_ for ABTS radical scavenging activity was markedly lower for the WildAO extract (67.33 ± 0.97 μg/mL) compared with the LabAO extract (86.86 ± 2 μg/mL). For DPPH radical scavenging activity, the difference was less pronounced, with an EC_50_ of 82.65 ± 1.03 μg/mL for WildAO extract and 86.46 ± 1.8 μg/mL for LabAO extract.

### 3.5. Mineral Composition

The macro element analysis revealed that LabAO contained significantly higher Mg (201.5 ± 10.5 mg/100 g DW) and Ca (40.01 ± 3.8 mg/100 g DW) compared with WildAO (Mg: 78.44 ± 4.5; Ca: 22.96 ± 4.5 mg/100 g DW) (Table 1). However, WildAO has higher K content (274.14 ± 14.4 mg/100 g DW) compared with LabAO (237.91 ± 11.4 mg/100 g DW). The Na content was remarkably higher for LabAO (185.86 ± 9.8 mg/100 g DW) than WildAO (37.14 ± 4.7 mg/100 g DW). The Na/K ratio was 0.14 for WildAO, much lower than the ratio for LabAO (0.78). The micro elements displayed an interesting pattern, notably Fe (0.48 mg/100 g DW) and Mn (0.03 mg/100 g DW) contents for LabAO were different than Fe (0.28 mg/100 g DW) and Mn (0.33 mg/100 g DW) contents of WildAO. Trace elements V, Cr, Co, Ni, As and Cd were present at very low concentrations (<0.1 mg/100 g DW), while Hg and Pb were not detected in WildAO or LabAO. In brief, lab-acclimatized *A. orientalis* (LabAO) showed higher contents of most macro elements except potassium, resulting in a less favorable Na/K ratio compared to the wild-type (WildAO) seaweed.

### 3.6. Non-Targeted Metabolites Profiling

**Figure 5:**
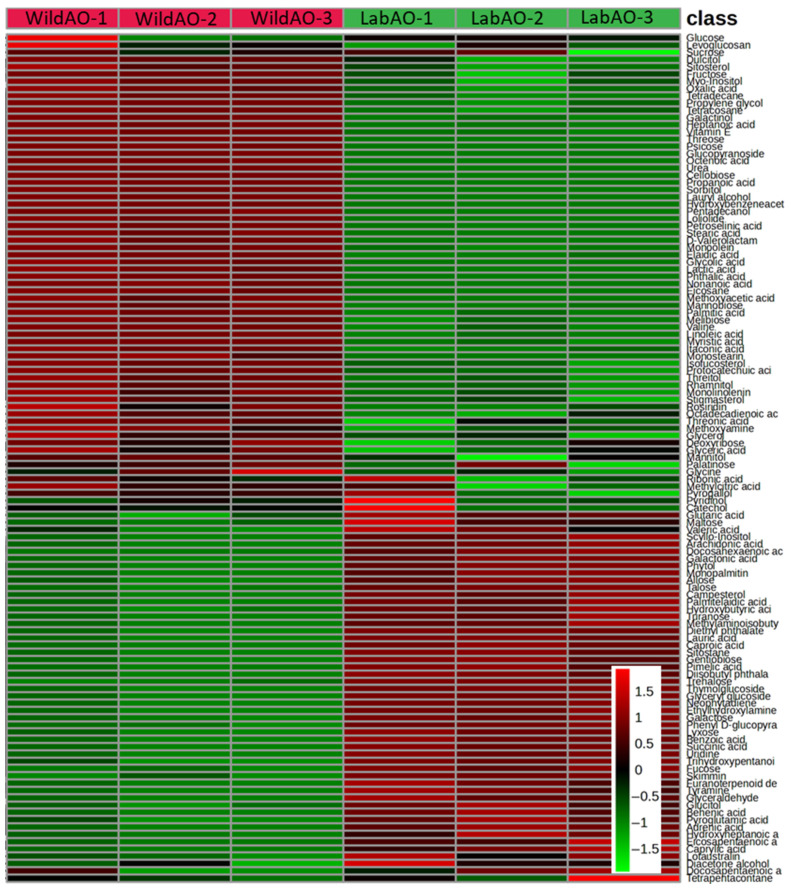
Metabolite heatmap of GC–MS non-targeted metabolite profiling of the LabAO and WildAO extracts of *A. orientalis*.

The GC–MS analysis of whole WildAO and LabAO biomass revealed distinct metabolite profiles (Figure 5, Supplementary Table S2). Lauryl alcohol and pentadecanol (alcohol derivatives) and eicosane (an aliphatic hydrocarbon) were detected in WildAO but not in LabAO. Conversely, phytol (alcohol derivative) was found in LabAO (0.61 ± 0.08 µg/100 mg) but not in WildAO. Tetracosane and tetradecane (aliphatic hydrocarbons) were present in both biomasses, with a significantly higher tetracosane content in WildAO (11.94 ± 0.19 µg/100 mg) compared with LabAO (3.53 ± 0.8 µg/100 mg). Among amino acids, valine was more abundant in WildAO (5.23 ± 0.25 µg/100 mg), while pyroglutamic acid was higher in LabAO (1.4 ± 0.28 µg/100 mg). Tyramine and methylaminoisobutyric acid were detected only in LabAO. Catechol and pyrogallol (aromatic compounds) were more abundant in LabAO. For disaccharides, melibiose and sucrose were more abundant in WildAO (20.02 ± 0.57 µg/100 mg and 27.06 ± 1.31 µg/100 mg, respectively), while trehalose and turanose were higher in LabAO (4 ± 0.15 µg/100 mg and 4.66 ± 1 µg/100 mg, respectively). Several fatty acids showed differential accumulation. Heptanoic and myristic acids were more abundant in WildAO. Galactinol (a glycoside) was significantly higher in WildAO (29.73 ± 1.25 µg/100 mg), while skimmin and phenyl D-glucopyranoside were enriched in LabAO. Benzoic and succinic acids (organic acids) were found only in LabAO. Vitamin E was considerably higher in WildAO (43.94 ± 0.74 µg/100 mg) compared with LabAO (18.25 ± 0.9 µg/100 mg). Lyxose and allose (sugars) and campesterol and stigmasterol (steroids) also showed higher levels in LabAO.

### 3.7. Fatty Acid Profiling

The fatty acid methyl ester (FAME) analysis of the whole biomass revealed variations in lipid composition of LabAO and WildAO (Table 2). For LabAO, the most abundant fatty acid was arachidonic acid (450.76 ± 25.67 µg/gm), while undecylic acid (3.98 ± 0.33 µg/gm) was the least abundant. In WildAO, oleic acid was the most abundant (144.93 ± 11.08 µg/gm) and undecylic acid was not detected. Several fatty acids, including myristoleic acid, pentadecylic acid, palmitoleic acid, heptadecanoic acid, palmitic acid, oleic acid, linoleic acid, and docosahexaenoic acid (DHA), were present at considerably higher levels in WildAO. In contrast, arachidonic acid was substantially more abundant in LabAO. The FAME analysis of whole biomass revealed distinct lipid profiles influenced by the growth environment; wild-type *A. orientalis* showed abundance of beneficial fatty acids like DHA, while the lab-acclimatized was notably enriched in arachidonic acid.

### 3.8. Amino Acid Composition

The amino acid profiling (µg/100 mg DW) by HPLC revealed distinct compositions of LabAO and WildAO (Table 3). Among the essential amino acids, phenylalanine was the most abundant in WildAO (493.01 ± 32.26 µg), while methionine was the most abundant in LabAO (62.27 ± 4.05 µg). Conversely, LabAO showed notably higher levels of isoleucine (58.41 ± 4.82 µg) and methionine compared with WildAO. In contrast, WildAO had substantially higher levels of histidine (11.62 ± 2.23 µg), lysine (25.33 ± 2.10 µg), phenylalanine (493.01 ± 32.26 µg), and valine (47.16 ± 3.97 µg). Among the non-essential amino acids, aspartic acid (338.09 ± 21.37 µg), serine (46.3 ± 3.15 µg), glycine (8.61 ± 1.82 µg), and alanine (40.56 ± 5.65 µg) were higher in WildAO. Conversely, glutamic acid (12.54 ± 3.51 µg), arginine (13.68 ± 1.86 µg), proline (31.04 ± 4.43 µg), and cysteine (115.89 ± 15.03 µg) were more abundant in LabAO. Overall, the two *A. orientalis* types exhibited significantly different amino acid profiles, with wild-type generally having higher levels of several essential and non-essential amino acids, notably phenylalanine and aspartic acid, while lab-acclimatized was rich in isoleucine, methionine, proline, and cysteine.

### 3.9. Antiproliferative Activity and Nuclear Fragmentation Assay

**Figure 6:**
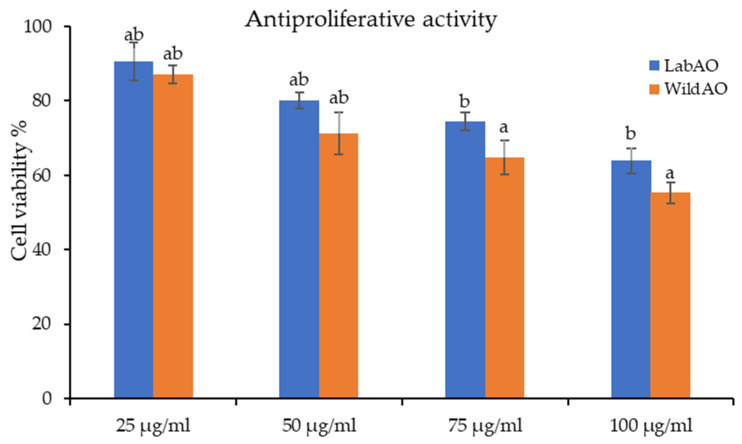
Cell viability percentages of the HeLa cell line after treatment with the LabAO and WildAO extracts. Data are expressed as mean ± SEM (*n* = 3), and different letters indicate a statistically significant difference (*t*-test *p* < 0.05).

The impact of varying concentrations (25, 50, 75, and 100 µg/mL extract) of WildAO and LabAO extracts were evaluated on HeLa cancer cells using an MTT assay (Figure 6). At the highest tested concentration (100 µg/mL), cell viability decreased to 63.93 ± 3.4% for the LabAO extract and to 55.25 ± 2.8% for the WildAO extract. The strong linearity of the dose–response relationship (R^2^ = 0.95–0.99) enabled reliable estimation of the EC_50_: 153.35 ± 10.18 µg/mL for LabAO and 118.63 ± 14.14 µg/mL for WildAO extract. These results indicate that both extracts exert dose-dependent antiproliferative effect, with the WildAO extract showing greater potency. 

**Figure 7:**
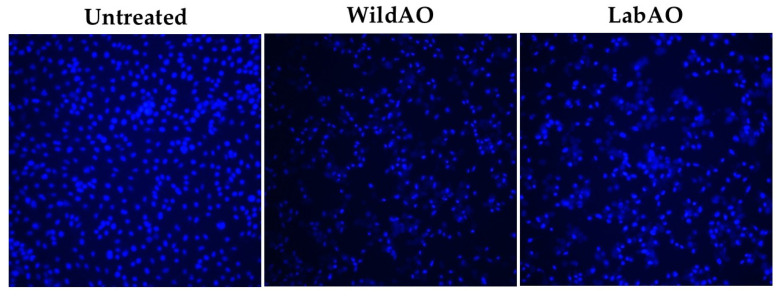
Nuclear staining of HeLa cell line after treatment with EC_50_ dose of the LabAO or WildAO extracts.

Apoptotic changes were further assessed using Hoechst 33342 staining at the respective EC_50_ (Figure 7). Cells treated with the WildAO extract exhibited pronounced nuclear condensation and chromatin fragmentation, indicative of apoptosis. In contrast, cells treated with LabAO extract showed fewer apoptotic features, although there was some degree of nuclear condensation.

### 3.10. Soft Gel Agar Assay

**Figure 8:**
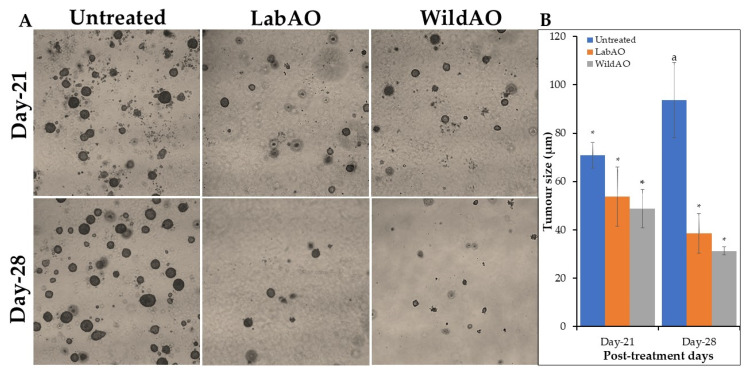
Soft gel agar assay to study antitumorigenic property of the LabAO and WildAO extracts on HeLa cells treated with EC_50_. (**A**) Figure represents the image representation of colony formation at 21st and 28th day intervals. (**B**) Graphical presentation changes in the colony/tumor size of untreated and treated cells. Different letters indicate a statistically significant difference (*t*-test, *p* < 0.05), and ‘*’ shows no significant difference.

The antitumorigenic potential of the extracts was evaluated using soft agar assay by monitoring colony growth over 28 days (Figure 8). Untreated control cells displayed continued proliferation, with the colony size increasing from 70.89 ± 5.38 µm on day 21 to 93.7 ± 15.48 µm on day 28. In contrast, both extracts significantly inhibited anchorage-independent growth. Cells treated with LabAO extract showed a reduction in colony size from 53.75 ± 12.34 µm (day 21) to 38.49 ± 8.22 µm (day 28), while cells treated with WildAO extract exhibited a more pronounced decrease from 48.71 ± 8.01 µm on day 21 to 31.21 ± 1.62 µm on day 28. Overall, both extracts effectively suppressed HeLa cell proliferation and tumorigenic potential, with WildAO extract demonstrating slightly greater inhibitory activity.

### 3.11. Oxidative Stress and Oncogene Expression Study

**Figure 9:**
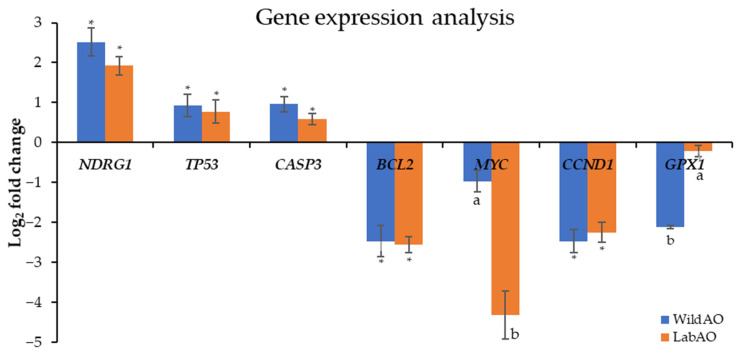
Gene expression log_2_-fold change of oxidative and cancer-linked key genes in HeLa cell line treated with EC_50_ of the LabAO or WildAO extracts. Different letters indicate a statistically significant difference (*t*-test, *p* < 0.05) and ‘*’shows no significant difference.

Gene expression analysis (log_2_ fold-change) revealed distinct effects of the WildAO and LabAO extracts on HeLa cells (Figure 9). Both extracts upregulated the tumor suppressor gene *NDRG1*, with the WildAO extract exerting a stronger effect (2.52 ± 0.35) compared with the LabAO (1.92 ± 0.23). Similarly, the proapoptotic genes *TP53* and *CASP3* were upregulated by both extracts, with the WildAO extract showing higher fold-change (*TP53*: 0.92 ± 0.28; *CASP3*: 0.96 ± 0.19) than the LabAO (*TP53*: 0.77 ± 0.29; *CASP3*: 0.58 ± 0.14). The antiapoptotic gene *BCL2* was downregulated to a similar extent by the WildAO (−2.47 ± 0.39) and LabAO (−2.56 ± 0.2) extracts. *MYC*, an oncogene, was strongly suppressed by the LabAO extract (−4.32 ± 0.7), more so than by the WildAO extract (−0.97 ± 0.27). The cell cycle regulator *CCND1* was downregulated by both extracts (WildAO: −2.47 ± 0.29; LabAO: −2.25 ± 0.25), while the antioxidant gene *GPX1* exhibited a greater reduction with the WildAO (−2.12 ± 0.04) than with the LabAO (−0.22 ± 0.14). In summary, both extracts modulated key cancer-related genes: The WildAO extract more strongly enhanced tumor suppressor and proapoptotic pathways, whereas the LabAO extract produced a more pronounced downregulation of the oncogene *MYC*.

## 4. Discussion

Growth analysis indicated that laboratory acclimatization of *A. orientalis* maintains stable growth and metabolic efficiency under controlled conditions, supporting its suitability for cultivation. However, the DGR of *Ulva intestinalis* (7.13 ± 3.44%) was higher than that of LabAO (2.71 ± 0.21%) [42]. Similarly, *Ulva* spp. have been reported to have achieve a substantially higher DGR (33 ± 6%) under nutrient-rich conditions [43]. The reported relative growth rates for *Ulva prolifera* (0.81 ± 0.098) and *Ulva meridionalis* (1.41 ± 0.081) further highlight interspecies variability [44]. In this study, the relatively low respiration rate (5.34 ± 0.17 mg O_2_ L^−1^ day^−1^) compared to GPP (12.55 ± 0.1 mg O_2_ L^−1^ day^−1^) indicates efficient carbon assimilation, which may explain the higher sugar content and TPC in LabAO. 

The higher WSC and WHC observed in LabAO suggest structural modifications in the polysaccharide matrix due to controlled cultivation conditions, potentially resulting in more porous or hydrophilic cellular structures. The WSC of WildAO and LabAO (5 ± 0.21 and 6 ± 0.25 mL/g DW, respectively) is lower than that of *Ulva lactuca* (11.2 ± 0.57 mL/g) [45], reflecting a higher capacity to absorb water and swell. In another study, WSC of *Caulerpa racemosa* and *Halimeda macroloba* (2.16  ±  0.57 and 2.42  ±  0.12 mL/g DW, respectively) was higher than that of WildAO and LabAO [46]. Those species also had a higher WHC than the samples from the present study: 6.56  ±  0.28 g/g for *C. racemosa* and 6.32  ±  0.14 g/g for *H. macroloba* [46]. The WHC of seaweed is associated with the cell wall and also influenced by temperature, pH, and ionic strength [47]. The OHC of WildAO (1.8 ± 0.12 g/g) was slightly higher than that of *C. racemosa* (1.15  ±  0.05 g/g) and *H. macroloba* (1.28  ±  0.22 g/g) [46]. These values are substantially lower than that reported in another study for *A. orientalis* (8.00 ± 0.002 g/g), *Ulva fasciata* (7.00 ± 0.02 g/g) and *U. lactuca* (7.00 ± 0.03) [23]. These variations demonstrate that cultivation conditions strongly influence physicochemical properties, which are critical for industrial applications requiring specific hydration or oil-binding characteristics. 

Biochemical analysis revealed that LabAO had higher total sugar content and TPC, suggesting that controlled cultivation conditions favor carbohydrate accumulation and biosynthesis of phenolic compounds, possibly due to optimized nutrient availability and reduced environmental stresses. The total sugar content in LabAO and WildAO (47.11 ± 0.52 & 43.8 ± 3.76% Glc eq. DW, respectively) was higher than those reported for *C. racemosa* (38.18%) and *Caulerpa entilifera* (13.63%) [48]. There were lower total sugar contents reported in *U. lactuca* (24.53% ± 0.04) and *Caulerpa macrodisca* (34.57 ± 2.12%) [49,50]. Choudhary et al. reported a higher TPC (107 ± 1 mg g^−1^) for than for Wild AO and LabAO (41.53 ± 1.6 and 51.28 ± 0.54 mg GAE/g, respectively) [23]. Another study reported a much lower TPC for *U. lactuca* and *U. compressa* (5.61 ± 4.13 and 0.66 ± 0.03 mg GAE/g, respectively) compared with WildAO and LabAO [49,51]. In contrast, the elevated TFC and protein content in WildAO likely reflect adaptive responses to natural stressors such as exposure to ultraviolet light, temperature fluctuations, and microbial interactions. The TFC of WildAO (123.62 ± 2.97 mg Q/g) is much higher than that of *U. intestinalis* (7.92 ± 1.92 mg Q/g DW) but lower than that of *A. orientalis* (277 ± 3 mg g^−1^) [23,52]. The protein content in both WildAO and LabAO is bit lower (5.9 ± 0.32 µg BSA eq/mg DW) than that of observed in *C. lentillifera* and *C. racemosa* (0.53–10.5% DW) [53,54]. Low chlorophyll content in LabAO suggested a reduced photosynthesis under controlled light conditions. However, pigment analysis showed a higher chlorophyll content in WildAO (8.81 ± 0.58 mg/L) compared with LabAO and *U. prolifera* (0.805 mg/gm). WildAO also had a higher carotenoid content (1.63 ± 0.08 mg/L) compared with *U. prolifera* (0.086 mg/gm) [55]. 

The ash content of LabAO and WildAO ranged from 37.9% to 42.7%, which is higher than that of *C. racemosa* (34.44%) but lower than that of *C. lentilifera* (63.83%) [48]. Another study reported that *C. fragile* exhibited higher ash content (66.8%) than WildAO [56]. Hiraoka et al. reported an ash content of 14.75 ± 0.74% in *U. prolifera*, which is substantially lower than the values observed in WildAO and LabAO [44]. The lower ash content in LabAO implies reduced mineral uptake compared with WildAO, which accumulates more inorganic matter from its natural habitat. The elevated ash content observed in the present study indicates both the diversity of mineral composition [57] and the presence of salt within the leaves [58]. The significantly higher moisture content in WildAO likely reflects natural hydration patterns in marine ecosystems, whereas the reduced moisture in LabAO may result from standardized drying protocols or intrinsic physiological adaptation developed under controlled conditions. The moisture content in WildAO and LabAO ranged from 61.04% to 72.51%, considerably higher than that reported for *Ulva* spp. (40–47%) [51]. These differences underscore the complex interplay between environmental factors and the biochemical composition of *A. orientalis*, which may influence its potential applications.

The ABTS and DPPH radical scavenging activity (EC_50_) of WildAO and LabAO extracts ranged from 65 to 85 ug/mL. The WildAO extract had lower EC_50_ values (ABTS: 67.33 ± 0.97; DPPH: 82.65 ± 1.03 µg/mL extract) compared to LabAO (ABTS: 86.86 ± 2; DPPH: 86.46 ± 1.8 ug/mL extract). These results suggest that WildAO extract contains phenolic compounds capable of acting as antioxidants through free radical scavenging activity. The superior DPPH scavenging of the WildAO extract at lower concentrations (25–50 μg/mL extract) correlates with its TFC (123.62 mg Q g^−1^ extract) and protein content (5.11 µg BSA eq/mg DW), indicating that these components contribute significantly to hydrogen-donating antioxidant capacity. Conversely, the initially stronger ABTS radical scavenging activity of the LabAO extract may result from its higher TPC (51.28 mg GAE g^−1^ extract) and total sugars content (47.11% Glc eq. DW), which are known to effectively scavenge cationic radicals. Studies on other seaweeds have revealed higher half-maximal inhibitory concentrations (IC_50_) for DPPH and ABTS radical scavenging: *Chaetomorpha linum* (DPPH: 1.09 mg/mL), *U. lactuca* (ABTS: 1.35 mg/mL), and *C. linum* (ABTS: 1.49 mg/mL) [59]. In a study on green seaweeds, the ABTS IC_50_ was <1 mg/mL for *A. orientalis*, 10.0 ± 1.5 mg/mL for *Caulerpa scalpelliformis*, 7.0 ± 0.2 mg/mL for *U. fasciata*, and 1.0 ± 0.1 mg/mL for *U. lactuca*. The corresponding DPPH IC_50_ values were 2.0 ± 0.1, 4.0 ± 0.1, 9.0 ± 1.0, and 1.0 ± 0.2 mg/mL, respectively [23]. *U. prolifera* exhibited a low IC_50_ for DPPH (43.52 μg/mL) and ABTS (54.10 μg/mL) radical scavenging [60]. Collectively, these results highlight the superior antioxidant potential of WildAO, particularly due to its TFC and protein content.

Macro elements (Na, Mg, Ca, K) varied significantly depending on the growth environment. LabAO had higher Na, Mg, and Ca contents, likely reflecting controlled nutrient supply. Micro elements (B, Fe, Mn, Al) also differed, suggesting variations in metabolic uptake or bioavailability. The low Cr content in LabAO and variations in trace minerals such as strontium (Sr) further demonstrate environmental influence on elemental accumulation. The Na/K ratio was 0.14 for WildAO and 0.78 for LabAO, indicating that WildAO may be more desirable for consumers seeking lower sodium intake relative to potassium. Comparatively, *Ulva rigida* showed higher micro element content (Na: 3604 ± 384 to 4700 ± 290 mg/100 g DW; Mg: 2695 ± 241 to 3992 ± 196 mg/100 g DW; K: 2157 ± 204 to 3460 ± 336 mg/100 g DW; Ca: 387 ± 37 to 498 ± 71 mg/100 g DW) than WildAO and LabAO. Seasonal and environmental factors likely contribute to such variations [61]. Similarly, *U. lactuca* exhibited lower mineral contents during summer (Ca: 97.8 ± 0.56 mg/100 g; Na: 11 ± 0.36 mg/100 g; K: 5.34 ± 0.37 mg/100 g; Mg: 9 ± 0.22 mg/100 g) [62]. These findings highlight that growth environment impacts mineral composition, which may influence nutritional suitability.

Metabolomic analysis revealed distinct biochemical compositions between WildAO and LabAO. Certain metabolites were exclusively to one growth type, indicating the influence of the environment on metabolic pathways. LabAO had lower levels of some alcohols and hydrocarbons, suggesting altered lipid metabolism. WildAO presented higher concentration of valine, melibiose, and vitamin E, potentially reflecting adaptation to environmental stressors. LabAO contained pyroglutamic acid, trehalose, turanose, caproic acid, docosahexaenoic acid, skimmin, and certain organic acids, indicating different metabolic priorities under controlled conditions. Tanna et al. previously reported about 47 compounds in *A. orientalis*, including sugars (sucrose: 1830 µg g^−1^; maltose: 1430 µg g^−1^), tricarboxylic acid cycle metabolites (malic acid: 175 µg g^−1^; citric acid: 5 µg g^−1^; succinic acid: 4 µg g^−1^), and fatty acids (palmitic acid: 57 µg g^−1^; stearic acid: 50 µg g^−1^) by non-targeted GC–MS metabolites profiling [3]. Similar profiling in *U. lactuca* identified fatty acids and its derivatives like arachidonic acid, linolenic acid and stearic acid; glycerides, sterols, oxysterols, cermides, terpenoids, carbohydrates and amino acids [63]. These results underscore the metabolic plasticity of *A. orientalis* and highlighting the environmental impact on biochemical composition.

**Figure 10:**
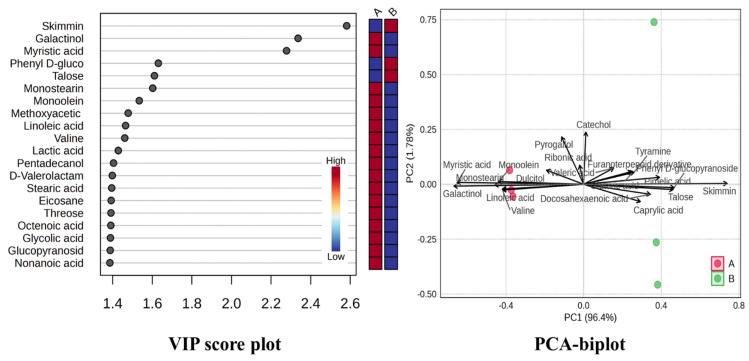
PL-SDA (Q^2^ ≥ 0.9, and Q^2^–R^2^ ≤ 0.1) biplot and VIP score plot. A and B represent WildAO and LabAO specimens, respectively.

The PCA biplot and VIP score plot demonstrated clear metabolic separation between WildAO and LabAO, with PC1 accounting for 96.4% of variance (Figure 10). LabAO was associated with key metabolites, such as skimmianine, galactinol, myristic acid, talose, and monoolein, with high VIP scores (>2.4). WildAO showed stronger associations with galactinol, linoleic acid, valine, and dulcitol. These metabolite shifts reflect distinct metabolic reprogramming under the lab adaptation. PLS-DA modeling showed robustness and statistical significance (Q^2^ ≥ 0.9, and Q^2^–R^2^ ≤ 0.1; *n* = 100), confirming the predictive power and identifying metabolites discriminating between environmental conditions. Results highlight the usefulness of metabolite biomarkers for monitoring environmental or physiological adaptation in *A. orientalis*.

**Figure 11:**
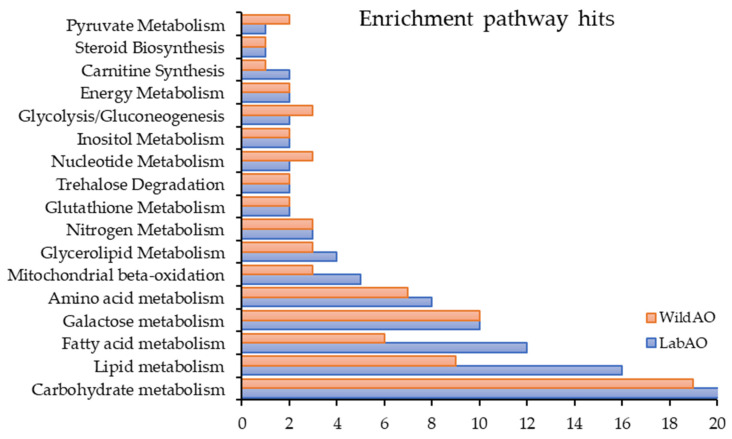
Comparative enrichment analysis generating metabolic pathway hits of the LabAO and WildAO extracts using metaboanalyte.

MetaboAnalyst pathway analysis provides useful insights into the differential metabolic activity between LabAO and WildAO (Figure 11). Higher lipid and fatty acid metabolism in LabAO reflects optimized nutrient availability and lower environmental stress. Tyrosine metabolism and phytanic acid oxidation pathways were also prominent in LabAO. Conversely, WildAO exhibited slightly higher nucleotide and pyruvate metabolism, potentially reflecting higher energy demands or environmental stress responses. These results suggest that environmental conditions strongly influence metabolic pathway prioritization.

FAME analysis revealed that WildAO had higher levels of several fatty acids, including DHA, while LAbAO had elevated arachidonic acid. A comparative study reported the following fatty acids in *U. lactuca*: palmitic acid (6.20 ± 0.32 µg/mg), stearidonic (1.04 ± 0.02 µg/mg), palmitoleic (0.99 ± 0.03 µg/mg), and behenic (0.91 ± 0.03 µg/mg) [64], while another study reported that *U. intestinalis* had a low saturated fatty acid content (13.7% of total fatty acids) and a high monounsaturated fatty acid content (36.97% of total fatty acids) [65]. These variations in fatty acids could affect the nutritional value and potential applications of *A. orientalis*, depending on its habitat.

The distinct amino acid profiles observed between WildAO and LabAO highlight the significant impact of the cultivation environment on the biochemical synthesis and accumulation within the seaweed. The higher concentrations of essential amino acids such as phenylalanine, lysine, and valine in WildAO may confer adaptive advantages in natural habitats, potentially reflecting a broader metabolic capacity or a heightened response to diverse environmental stimuli [66]. For example, phenylalanine serves as a precursor for various secondary metabolites, which may be upregulated under environmental stress conditions in the wild [67]. Similarly, the markedly elevated levels of aspartic acid and alanine in WildAO suggest active nitrogen metabolism pathways adapted to natural environmental conditions [68]. In contrast, the increased levels of isoleucine, methionine, proline, and cysteine in LabAO indicate specific metabolic adjustments under controlled conditions. Proline accumulation is commonly associated with osmotic stress tolerance and, even in a laboratory setting, may reflect a baseline stress response or a targeted growth strategy [69]. Cysteine, a sulfur-containing amino acid, plays a critical role in antioxidant defense, suggesting that lab-acclimatized seaweed may possess enhanced capacity to manage oxidative processes [70]. The overall amino acid profile, characterized by low leucine and high phenylalanine levels, may represent species-specific metabolic traits of green seaweed. The amino acid composition in macroalgae is known to vary substantially depending on species, environmental conditions, and physiological state. Aromatic amino acids, such as phenylalanine, are often elevated due to their roles in the shikimate pathway and in the biosynthesis of phenolic and other secondary metabolites, whereas branched-chain amino acids like leucine fluctuate according to protein composition and nitrogen metabolism within algal tissue [71,72,73]. These differences in amino acid profiles suggest that wild and lab-acclimatized *A. orientalis* may offer distinct health benefits. Such variations could inform the use of this seaweed in functional food and nutritional applications. 

The MTT assay results demonstrated that both the WildAO and LabAO extracts exerted dose-dependent cytotoxicity against HeLa cells. The WildAO extract exhibited a lower EC_50_ (118.63 ± 14.14 µg/mL extract) than the LabAO extract (153.35 ± 10.18 µg/mL extract), indicating higher efficacy. Similarly, previous studies on *U. rigida* have shown a dose-dependent cytotoxic effect on HepG2 cells, with maximal inhibition observed at 1000 μg/mL [74]. The differential efficacy between the WildAO and LabAO extracts likely reflects variation in the concentrations of bioactive metabolites, as indicated by metabolite profiling. Morphological observations revealed condensed chromatin and nuclear fragmentation in WildAO-extract-treated cells, consistent with apoptosis induction [75]. LabAO-extract-treated cells displayed fewer apoptotic changes at their respective EC_50_, although some nuclear damage was still evident.

The soft agar assay further supported the antitumorigenic potential of both WildAO and LabAO extract against HeLa cells. Treated cells exhibited a reduction in colony size compared to the continuous growth observed in untreated controls, indicating inhibition of anchorage-independent proliferation, a hallmark of transformed cells [76]. The WildAO extract consistently demonstrated a slightly greater capacity to reduce colony size at days 21 and 28, suggesting a higher concentration or more potent combination of bioactive compounds [77]. Overall, both extracts effectively suppressed HeLa cell tumorigenesis, with the WildAO extract producing marginal strong inhibitory effect.

Gene expression analysis revealed that both WildAO and LabAO extracts could modulate pathways relevant to cancer progression. The upregulation of tumor suppressor genes, including *NDRG1* and *TP53*, suggests activation of cellular defense mechanisms [78]. Concurrent upregulation of *CASP3*, a critical effector in apoptosis, further supports a proapoptotic effect [79]. Both extracts also downregulated the antiapoptotic gene *BCL2*, promoting a shift toward programmed cell death [80]. Additionally, significant downregulation of the oncogene *MYC*, particularly by the LabAO extract, indicates strong inhibition of cell proliferation [81], while suppression of *CCND1* suggests potential cell cycle arrest [82]. The differential downregulation of *GPX1*, an antioxidant enzyme encoding gene, points to alteration in the cellular redox state, which can influence apoptosis and other cellular processes [83]. Collectively, these findings indicate that both wild-type and lab-acclimatized *A. orientalis* extracts can modulate cancer-associated pathways by enhancing natural defense mechanisms and promoting apoptosis.

## 5. Conclusions

This comparative analysis of WildAO and LabAO provides valuable insights for the sustainable cultivation of *A. orientalis* and its potential applications. LabAO demonstrates the ability to maintain balanced growth and metabolic efficiency, supporting the suitability of *A. orientalis* for controlled cultivation. In contrast, WildAO often exhibits higher levels of stress-response metabolites, such as flavonoids and proteins, reflecting the adaptation of *A. orientalis* to natural environmental stressors. Nevertheless, LabAO can accumulate substantial amount of beneficial compounds, including sugars and phenolics, under optimized conditions. The observed differences in physicochemical properties, such as water- and oil-holding capacities, as well as distinct fatty acid and amino acid profiles, highlight the significant influence of the cultivation environment on the functional attributes of the biomass. For example, the superior Na/K ratio in WildAO makes it nutritionally more advantageous for sodium-conscious consumers.

For industrial-scale production, controlled cultivation of *A. orientalis* offers a way to obtain a sustainable and consistent biomass supply, enabling optimization of growth conditions to enhance specific desirable compounds, such as sugars and phenolic compounds. However, naturally harvested *A. orientalis* demonstrates comparable anticancer and antioxidant properties, making it particularly suitable for nutraceuticals applications or high-value antioxidant products. Controlled cultivation strategies can also help to reduce dependency on wild harvesting, supporting environmental sustainability. While the current study demonstrates promising bioactivity in crude extracts, further investigations are necessary to validate these effects, particularly on non-cancerous (normal) cell lines, before strong nutraceutical claims can be made. Future research should focus on fully exploring the potential of *A. orientalis* as a functional food or nutraceutical resource.

## Figures and Tables

**Table 1:** Mineral/elemental compositions of the LabAO and WildAO samples of *A. orientalis*. Results are presented as the mean ± SEM (*n* = 3), with distinct letters denoting significant differences (*p* < 0.05), as determined by *t*-test.

Minerals	Wild Type	Lab Type
Macro element		
Na	37.14 ± 4.7 ^a^	185.86 ± 9.8 ^b^
Mg	78.44 ± 4.5 ^a^	201.5 ± 10.5 ^b^
P	0.05 ± 0 ^a^	0.31 ± 0 ^b^
K	274.14 ± 14.4 ^ab^	237.91 ± 11.4 ^ab^
Ca	22.96 ± 14.4 ^ab^	40.01 ± 3.8 ^ab^
Micro elements		
B	2.37 ± 0.9 ^ab^	3.47 ± 1.3 ^ab^
Mn	0.33 ± 0 ^b^	0.03 ± 0 ^a^
Fe	0.28 ± 0 ^a^	0.48 ± 0 ^b^
Co	0.003 ± 0 ^b^	0.002 ± 0 ^a^
Ni	0.009 ± 0 ^b^	0.001 ± 0 ^a^
Heavy elements		
Cr	0.009 ± 0 ^b^	0.002 ± 0 ^a^
As	-	0.002 ± 0
Cd	0.001 ± 0	0.001 ± 0
Hg	-	-
Pb	-	-
Trace elements		
Li	0.03 ± 0 ^b^	0.007 ± 0 ^a^
Rb	0.08 ± 0 ^b^	0.05 ± 0 ^a^
Sr	2.15 ± 0 ^a^	7.41 ± 0.5 ^b^
V	0.001 ± 0 ^a^	0.06 ± 0.0 ^b^

-: Below the limit of quantitation (LOQ). The quantitative data are expressed in mg/100 g dry weight (DW).

**Table 2:** Fatty acid composition (µg/gm) of the LabAO and WildAO specimens of *A. orientalis*.

Fatty Acids	Chain Length	Lab Type	Wild Type
Caprylic acid	C8:0	17.81 ± 3.93 ^ab^	23.72 ± 1.08 ^ab^
Undecylic acid	C11:0	3.98 ± 0.33	-
Lauric acid	C12:0	17.25 ± 1.26 ^ab^	19.46 ± 1.56 ^ab^
Tridecylic acid	C13:0	3.02 ± 0.3	-
Myristoleic acid	C14:1	22.62 ± 2.51 ^a^	57.84 ± 3.8 ^b^
Pentadecylic acid	C15:0	36.38 ± 4.82 ^a^	94.22 ± 6.74 ^b^
Palmitic acid	C16:0	53.31 ± 7.51 ^ab^	75.82 ± 8 ^ab^
Palmitoleic acid	C16:1	15.51 ± 3.58 ^a^	67.7 ± 4.02 ^b^
Heptadecanoic acid	C17:0	20.69 ± 2.63 ^a^	92.94 ± 6.22 ^b^
Oleic acid	C18:1	105.67 ± 12.28 ^ab^	144.93 ± 11.08 ^ab^
Linoleic acid	C18:2	24.67 ± 3.61 ^a^	73.35 ± 6 ^b^
Arachidonic acid	C20:4	450.76 ± 25.67 ^b^	101.55 ± 17.27 ^a^
Eicosapentaenoic acid	C20:5	26.64 ± 6.24 ^ab^	18.07 ± 0.64 ^ab^
Docosahexaenoic acid	C22:6	28.18 ± 6.03 ^a^	86.56 ± 5.22 ^b^

-: Not detected. Results are presented as the mean ± SEM (*n* = 3), with distinct letters denoting significant differences (*p* < 0.05), as determined by *t*-test.

**Table 3:** Amino acid composition (in µg/100 mg DW) of the LabAO and WildAO extracts of *A. orientalis*.

Amino Acids	LabAO	WildAO
Essential amino acids		
Histidine	3.25 ± 2.52 ^a^	11.62 ± 2.23 ^b^
Threonine	17.09 ± 3.25 ^ab^	10.88 ± 2.87 ^ab^
Valine	7.86 ± 1.87 ^a^	47.16 ± 3.97 ^b^
Methionine	62.27 ± 4.05 ^b^	11.26 ± 3.93 ^a^
Isoleucine	58.41 ± 4.82 ^b^	10.47 ± 1.41 ^a^
Leucine	2.44 ± 0.35 ^ab^	4.22 ± 1.04 ^ab^
Phenylalanine	192.31 ± 7.18 ^a^	493.01 ± 32.26 ^b^
Lysine	10.04 ± 1.97 ^a^	25.33 ± 2.10 ^b^
Non-essential amino acids		
Aspartic acid	86.5 ± 8.24 ^a^	338.09 ± 21.37 ^b^
Glutamic acid	12.54 ± 3.51 ^ab^	10.42 ± 1.7 ^ab^
Serine	27.72 ± 4.47 ^a^	46.3 ± 3.15 ^b^
Glycine	1.58 ± 0.57 ^a^	8.61 ± 1.82 ^b^
Arginine	13.68 ± 1.86 ^b^	8.06 ± 1.47 ^a^
Alanine	2.44 ± 0.2 ^a^	40.56 ± 5.65 ^b^
Proline	31.04 ± 4.43 ^b^	3.79 ± 0.44 ^a^
Tyrosine	54.76 ± 12.16 ^ab^	48.67 ± 4.41 ^ab^
Cysteine	115.89 ± 15.03 ^ab^	80.71 ± 8.92 ^ab^

Results are presented as the mean ± SEM (*n* = 3), with distinct letters denoting significant differences (*p* < 0.05), as determined by *t*-test.

## Data Availability

Dataset available on request.

## Supplementary Materials

The following supporting information can be downloaded at: https://www.mdpi.com/article/10.3390/foods15071252/s1, Table S1: Primer sets and optimized PCR conditions for quantitative real-time PCR; Table S2: Non-targeted metabolites profiling.
