# Supplementary material for: Comparative Metabolite Profiling and Antiproliferative Characterization of Lab-Acclimatized and Wild Green Seaweed Acrosiphonia orientalis to Reveal Its Nutraceutical Potential
Source: Foods. 2026 Apr 6;15(7):1252. doi: 10.3390/foods15071252 (PMC13074114; doi:10.3390/foods15071252)
Supplement: Supplementary file 1 [file foods-15-01252-s001.zip › Supplementry Table S1.pdf]

Table S1: Primer sets and optimized PCR conditions for quantitative real-time PCR.

S. No.	Genes	Gene's name	NCBI Gene ID	Primer pair (5′ → 3′)		RT-PCR condition
				Forward	Reverse	
1.	*GAPDH*	Glyceraldehyde-3-phosphate dehydrogenase	2597	TGCAACCGGGAAGGAAATGA	GCCCAATACGACCAAATCAGAGA	Initial denaturation:<br>94 °C for 30 s<br>40 cycles:<br>95 °C- 5 s<br>60 °C- 30 s<br><br>Melt curve:<br>50 to 94 with 0.05°C increment
2.	*GPX1*	Glutathione peroxidase 1	2876	TATCGAGAATGTGGCGTCCC	TCTTGGCGTTCTCCTGATGC	
3.	*CCND1*	Cyclin D1	595	GATCAAGTGTGACCCGGACT	CTTGGGGTCCATGTTCTGCT	
4.	*MYC*	MYC proto-oncogene	4609	TGGAAAACCAGCCTCCCG	TTCTCCTCCTCGTCGCAGTA	
5.	*TP53*	Tumour protein p53	7157	GACACGCTTCCCTGGATTGG	GACGGCAAGGGGGACAGAA	
6.	*NDRG1*	N-myc downstream regulated 1	10397	GGTCCCATTCATCTCCCCC	TGCTGTCACCTGCCTAGT	
7.	*BCL2*	BCL2 apoptosis regulator	596	GGATAACGGAGGCTGGGATG	TGACTTCACTTGTGGCCCAG	
8.	*CASP3*	Caspase 3	836	GTGAGGCGGTTGTAGAAGAGTT	TTAACGAAAACCAGAGCGCC	

Reference:

Tanna et al., 2024, *Nutrients*, 16, 1222.

Tanna et al., 2020, *Molecular Biology Reports*. 47, 7403–7411.
