# Supplementary material for: Comparative Metabolite Profiling and Antiproliferative Characterization of Lab-Acclimatized and Wild Green Seaweed Acrosiphonia orientalis to Reveal Its Nutraceutical Potential
Source: Foods. 2026 Apr 6;15(7):1252. doi: 10.3390/foods15071252 (PMC13074114; doi:10.3390/foods15071252)
Supplement: Supplementary file 1 [file foods-15-01252-s001.zip › Supplementry Table S2.pdf]

Table S2: Non-targeted metabolites profiling

Metabolites	WildAO_1	WildAO_2	WildAO_3	LabAO_1	LabAO_2	LabAO_3
	A1	A2	A3	B1	B2	B3
Lauryl alcohol	2.47609137	2.54516547	2.71056486	0	0	0
Pentadecanol	0.19889502	0.2170945	0.25650003	0	0	0
Phytol	0	0	0	0.6780016	0.65099006	0.52420302
Eicosane	0.6170945	0.6780016	0.56145927	0	0	0
Tetracosane	11.7230428	12.03328	12.0852163	2.7279053	4.33868484	3.54893071
Tetradecane	0.2958908	0.30816729	0.31195758	0.2170868	0.22461096	0.24224611
Tetrapentacontane	0.16597056	0.17956575	0.16400002	0.2648986	0.4341376	0.29815058
Glycine	0.09165971	0.12482381	0.14400002	0.1642062	0.13858157	0.1483092
Methylaminoisobutyric acid	0	0	0	0.0896818	0.12870494	0.08342442
Pyroglutamic acid	0.35335526	0.2958908	0.30816729	1.7327683	1.24071694	1.2341565
Tyramine	0	0	0	0.4341376	0.29815058	0.50429171
Valine	5.07886886	5.52598984	5.0914506	1.1341565	0.76254243	0.7214765
Catechol	0.13787863	0.13787863	0.14587554	0.1287049	0.12847683	0.8968183
Hydroxybenzeneacetic acid	0.14470034	0.14972015	0.14400002	0	0	0
Pyrogallol	0.36315245	0.35985753	0.38764462	0.2476839	0.13830626	1.01122061
Cellobiose	0.56932848	0.60820754	0.61412445	0	0	0
Maltose	0.43329027	0.47771005	0.50977863	1.2652592	1.15779698	1.80405767
Mannobiose	2.46313385	2.48846596	2.84532329	1.2814021	1.35733321	1.22785524
Melibiose	20.4911066	20.192111	19.3770638	8.1916924	7.0443984	6.08989179
Palatinose	0.45389113	0.55106455	0.59943847	0.94829	0.5247292	0.61889552
Sucrose	25.7416825	27.0733533	28.3660959	46.530948	35.5531113	43.9704617
Trehalose	0.25102551	0.30315245	0.35985753	4.1389113	3.84106056	4.03258121
Turanose	0	0	0	4.0325812	5.83215251	4.13891129
Adrenic acid	0	0	0	0.9251898	0.71245118	0.62015611
Arachidonic acid	0	0	0	0.7639177	0.88828494	0.70042414
Behenic acid	0	0	0	3.5517425	2.20846172	2.49999912
Caproic acid	7.53858293	7.71800437	7.67105624	62.477035	49.9579491	50.1074921
Caprylic acid	0	0	0	0.8882849	1.27741557	0.53230206
Docosahexaenoic acid	0.46996042	0.52610213	0.52762798	5.6781671	6.74245927	4.00297357
Docosapentaenoic acid	0.46049651	0.40227353	0.40887796	0.8765021	0.9251898	0.71059128
Eicosapentaenoic acid	0	0	0	0.1665195	0.366216	0.16752613
Elaidic acid	2.91492641	3.18299688	3.0839515	0.9596365	1.05802602	0.88828494
Heptanoic acid	25.7224781	27.3358295	28.7639439	11.423115	10.7503844	10.6865536
Lauric acid	0	0	0	0.4604965	0.40227353	0.43096657
Linoleic acid	1.16395664	1.31988557	1.37715059	0.2422962	0.20251442	0.18484804
Octadecadienoic acid	0.26440138	0.20172213	0.21883073	0.0739329	0.16651954	0.09127939
Myristic acid	3.83190023	4.27645491	4.03848504	0.2393042	0.13672567	0.18994261
Octenoic acid	0.46052118	0.51734474	0.53949625	0	0	0
Nonanoic acid	0.33410747	0.40330805	0.32332561	0	0	0
Palmitelaidic acid	0	0	0	1.1060145	1.57860569	1.2304724
Palmitic acid	6.28703733	6.67228332	6.72100255	4.654539	4.27645491	4.03848504
Petroselinic acid	1.70840622	1.6431374	1.77857525	0.3366033	0.34693902	0.34527392
Stearic acid	0.31324587	0.28753189	0.35542147	0	0	0

Valeric acid	0.480857	0.3785236	0.35542147	1.635267	1.03395824	2.08396474
Galactinol	29.7460562	28.4706216	30.9874503	1.5001089	1.06411619	1.17514767
Gentiobiose	0	0	0	0.3882649	0.28753189	0.35542147
Glucopyranoside	1.60479993	1.78564262	1.87131658	0	0	0
Glyceryl glucoside	0	0	0	3.2752648	3.42051223	3.2538966
Skimmin	0.1456923	0.38597365	0.15662496	17.439091	20.8500679	18.7749943
Thymolglucoside	0	0	0	0.8677336	0.86394439	0.78824767
Phenyl D-glucopyranoside	1.21642603	1.33914957	1.01608097	23.907934	28.773679	21.3545646
Monolinolenin	1.73798751	1.3394986	1.78564262	1.1139895	0.77602949	0.90341719
Monoolein	1.44378958	1.3394986	1.49732957	0.1846804	0.21355874	0.22735455
Monopalmitin	0	0	0	0.3128159	0.31095774	0.22735455
Monostearin	0.52003017	0.7044704	0.32062903	0	0	0
Uridine	0	0	0	0.429729	0.46599299	0.48191188
Benzoic acid				1.2135587	1.21016751	1.41362363
Glutaric acid	0.7044704	0.50048828	0.90341719	3.7489712	4.08057376	5.59396124
Glyceric acid	4.02814342	3.8047011	4.32255702	4.9619315	5.59396124	4.19320684
Hydroxybutyric acid	3.76398324	3.95370089	4.24631987	12.13732	14.7737441	12.3201166
Hydroxyheptanoic acid	1.35837101	1.45085695	1.41030205	5.6336338	4.48838762	3.66801022
Itaconic acid	15.9491185	15.5071077	15.5062236	7.8580062	8.82811975	7.10201085
Lactic acid	0.79596316	0.72509039	0.60590134	0	0	0
Methylcitric acid	8.37702654	8.12257437	8.10765967	8.2660688	9.7928949	12.6538417
Methoxyacetic acid	2.4533022	1.74978525	2.47928576	0	0	0
Phthalic acid	0.30720938	0.29922788	0.27874085	0	0	0
Propanoic acid	2.47221757	2.58978592	2.55132399	0	0	0
Oxalic acid	0.50902474	0.49457786	0.42960011	0.1679881	0.21282227	0.22011407
Pimelic acid	1.64490445	1.60660265	1.67927408	28.743502	16.7126025	24.1966897
Succinic acid	0	0	0	2.3151936	2.47221757	2.58978592
Pyridinol	0.51053644	0.64490445	0.61501831	0.9113662	0.9267891	1.16851188
Ethylhydroxylamine	0	0	0	37.546357	43.5146028	37.5463574
Methoxyamine	0.1419385	0.15438867	0.12658843	0.1543887	0.15438867	0.12658843
Urea	0.71837002	0.75649346	0.76031253	0	0	0
Diacetone alcohol	0.97839331	1.3261888	0.96506949	2.2233705	2.19081141	2.97801164
Diethyl phthalate	0	0	0	1.2336145	1.13874878	1.19514311
Diisobutyl phthalate	0	0	0	3.6439072	3.00788273	3.71800418
Levoglucosan	4.74793916	4.86554196	4.94681553	7.7940048	7.31074808	6.92583223
Lotaustralin	0.30688021	0.33018624	0.29232922	0.7324832	1.09745407	1.21002034
Rosiridin	1.42890853	1.24923292	1.48952319	1.510535	1.63894656	1.47011328
Propylene glycol	5.35748728	5.69566192	5.68826254	0.9364306	1.43249426	1.24084402
Vitamin E	43.0814171	44.3464671	44.3983073	19.074223	17.2849021	18.3927421
D-Valerolactam	0.16735331	0.13528704	0.14999297	0	0	0
Protocatechuic acid	15.6381812	15.7656327	16.3671714	9.6233248	7.79027074	9.70673346
Campesterol	0	0	0	8.7790593	11.8691568	9.85646186
Isofucosterol	8.22549638	8.21261182	8.73203777	4.8844599	3.49311294	4.83145999
Sitostane	0	0	0	0.4701133	0.37105224	0.43428022
Sitosterol	0.2103944	0.1556026	0.16735331	0.068251	0.08693479	0.10975746
Stigmasterol	38.9245363	39.0249223	38.9757584	43.952154	33.5985889	39.3269998
Galactonic acid	0	0	0	28.77643	26.8612309	22.1015209

Glycolic acid	0.21476722	0.18100363	0.17231636	0	0	0
Trihydroxypentanoic acid	0.21476722	0.18100363	0.18319186	0.7821057	0.86189061	0.82122821
Ribonic acid	1.23441836	1.20340161	1.03374778	1.1438287	1.51312312	2.6983261
Threonic acid	1.32128494	1.43624467	1.35866167	1.6388396	1.28222389	0.93947852
Dulcitol	0.94991546	0.95506336	0.97017102	0.4157089	0.49617452	0.78192015
Glucitol	1.90849751	2.00423378	2.1383817	5.536181	4.58168435	4.78394057
Glycerol	7.67317718	7.76054157	7.98987486	11.033746	9.26709835	10.2067065
Mannitol	0.14810796	0.18100363	0.17231636	0.0959253	0.20663182	0.16901141
Myo-Inositol	1.41834598	1.4677276	1.47321075	0.9457749	1.28222389	1.22355009
Rhamnitol	10.3915857	9.86603895	10.8738648	7.6873766	6.24953846	7.12302979
Scyllo-Inositol	0	0	0	0.2066318	0.2647092	0.1798318
Sorbitol	1.42641707	1.46527952	1.51944379	0	0	0
Threitol	63.7618635	63.8838404	66.4231708	62.540573	52.3968719	55.9620674
Allose	2.66007859	2.33565805	2.38962368	17.907887	18.4940354	13.2662816
Deoxyribose	2.18562292	2.27602683	2.62184205	2.92551	3.46058297	2.48195526
Fructose	0.31505844	0.34046323	0.34813398	0.0704978	0.1626986	0.14993183
Fucose	0.19548578	0.25979944	0.20403725	1.6466897	2.27901449	2.05831743
Galactose	0.45095343	0.46653928	0.43730705	2.3150584	2.70655735	2.44664722
Glucose	3.49513363	1.48860622	1.55486984	3.4991831	3.07048716	3.33965958
Glyceraldehyde	0	0	0	0.2083224	0.20368803	0.28662687
Lyxose	4.19180373	3.96160861	3.89542283	22.101984	22.6936696	25.527188
Psicose	0.14993216	0.17340886	0.15841228	0	0	0
Talose	0.44390389	0.38643692	0.45095343	8.8955902	10.0224781	7.02280634
Threose	0.12964498	0.15208796	0.14689223	0	0	0
Loliolide	0.17340886	0.15841228	0.17553142	0	0	0
Furanoterpenoid derivative	0	0	0	0.5449548	0.44523115	0.72267246
Neophytadiene	0	0	0	0.3500548	0.38643692	0.35095343
